# Human Placenta Buffers the Fetus from Adverse Effects of Perceived Maternal Stress

**DOI:** 10.3390/cells10020379

**Published:** 2021-02-12

**Authors:** Lahari Vuppaladhadiam, Jeannette Lager, Oliver Fiehn, Sandra Weiss, Margaret Chesney, Burcu Hasdemir, Aditi Bhargava

**Affiliations:** 1 Center for Reproductive Sciences, University of California San Francisco, San Francisco, CA 94143, USA; lahari.vuppala2018@gmail.com; 2 Department of Obstetrics and Gynecology, University of California San Francisco, San Francisco, CA 94143, USA; Jeannette.Lager@ucsf.edu (J.L.); burcu@dreadnought.org (B.H.); 3 NIH West Coast Metabolomics Center, University of California Davis Genome Center, Davis, CA 95616, USA; ofiehn@ucdavis.edu; 4 Department of Community Health Systems, Stress and Depression Research Lab, University of California San Francisco, San Francisco, CA 94143, USA; Sandra.Weiss@ucsf.edu; 5 The Osher Center for Integrative Medicine, University of California San Francisco, San Francisco, CA 94143, USA; Margaret.Chesney@ucsf.edu

**Keywords:** birth outcomes, cortisol, hair steroids, perceived stress, mass spectrometry

## Abstract

Maternal stress during pregnancy is linked to several negative birth outcomes. The placenta, a unique pregnancy-specific organ, not only nourishes and protects the fetus but is also the major source of progesterone and estrogens. As the placenta becomes the primary source of maternal progesterone (P4) and estradiol between 6–9 weeks of gestation, and these hormones are critical for maintaining pregnancy, maternal stress may modulate levels of these steroids to impact birth outcomes. The objective was to test whether maternal perceived stress crosses the placental barrier to modulate fetal steroids, including cortisol, which is a downstream indicator of maternal hypothalamic–pituitary–adrenal (HPA) axis regulation and is associated with negative fetal outcomes. Nulliparous women, 18 years or older, with no known history of adrenal or endocrine illness were recruited during their third trimester of pregnancy at the University of California San Francisco (UCSF) Mission Bay hospital obstetrics clinics. Simultaneous measurement of 10 steroid metabolites in maternal (plasma and hair) and fetal (cord blood and placenta) samples was performed using tandem mass spectrometry along with assessment of the perceived stress score and sociodemographic status. While the maternal perceived stress score (PSS) and sociodemographic status were positively associated with each other and each with the body mass index (BMI) (*r* = 0.73, *p* = 0.0008; *r* = 0.48, *p* = 0.05; *r* = 0.59, *p* = 0.014, respectively), PSS did not correlate with maternal or fetal cortisol, cortisone levels, or fetal birth weight. Regardless of maternal PSS or BMI, fetal steroid levels remained stable and unaffected. Progesterone was the only steroid analyte quantifiable in maternal hair and correlated positively with PSS (*r* = 0.964, *p* = 0.003), whereas cord estradiol was negatively associated with PSS (*r* = −0.94, *p* = 0.017). In conclusion, hair progesterone might serve as a better marker of maternal stress than cortisol or cortisone and maternal PSS negatively impacts fetal estradiol levels. Findings have implications for improved biomarkers of stress and targets for future research to identify factors that buffer the fetus from adverse effects of maternal stress.

## 1. Introduction

Experiencing too much stress from negative life events or other stressors during pregnancy can have serious consequences for the pregnant mother and the developing fetus, including higher risk of preterm birth and low birth weight [1,2,3]. The placenta forms during pregnancy from fetal trophoblasts and serves as a temporary organ that creates the link between mother and fetus, providing the fetus with oxygen and nutrients necessary for survival. The placenta is a complex vascular, endocrine, and immune organ that supports fetal growth, removes waste products from the fetus, acts as a barrier to prevent infections reaching the fetus, and maintains maternal health [4,5]. Importantly, a normal effective placenta should thus buffer and protect the fetus from exposure to unwanted stressors and toxins. The placenta synthesizes and secretes steroid and peptide hormones, which, in turn, regulate maternal and fetal hormone synthesis by the endocrine organs [6]. The placenta is crucial for the development of the fetal pituitary–adrenocortical axis, responds to maternal cortisol levels, and forms part of the body’s stress response system. Excessive stress may result in dysregulation of placental biology, with potential effects on biochemical and metabolic pathways based on the degree of stress [7,8]. Problems with the placenta are known to lead to conditions such as gestational diabetes, preeclampsia, fetal growth restriction, preterm birth, and stillbirth [9,10,11,12,13,14,15]. But the mechanisms responsible for healthy versus problematic outcomes from stress are not completely understood.

Maternal stress during pregnancy is linked to several negative outcomes for mothers and their offspring. About 40% of preterm births are spontaneous and of unknown etiology. Nearly three quarters of women report experiencing at least one stressful event in the year before the delivery of their child, and 5–6% report experiencing ≥ 6 stressful events [16]. Cortisol has been used frequently to measure stress, although many studies have found no relationship between cortisol and perceived stress [17,18]. There are also conflicting findings regarding the efficacy of cortisol in predicting adverse birth outcomes [19,20]. Thus, improved markers of stress-related risk for adverse outcomes are needed. Although stress-induced glucocorticoid release is thought to be a primary driver by which maternal stress negatively impacts pregnancy outcomes, stress also stimulates the secretion of progesterone from the adrenal gland. Neuroactive steroid metabolites of progesterone can then modulate hypothalamic–pituitary–adrenal axis function [21]. However, the role of progesterone and its metabolites in relation to human stress and in mediating birth outcomes is not well understood [22].

Progesterone is a steroid sex hormone that plays an important role in pregnancy. Progesterone concentrations steadily increase with gestation age and range from 100 to 300 ng/mL at term [23]. In humans, before 6 weeks of gestation, the corpus luteum is the main source of progesterone, with a gradual shift to placental production. Beyond 10 weeks of gestation, the placenta is the main source of progesterone [24]. Withdrawal of progesterone as a result of removal of the corpus luteum within 4 weeks of gestation results in abortion [25]. In lutectomized and agonadal woman who become pregnant, exogenous progesterone administration helps maintain pregnancy until the end of first trimester, after which placental progesterone secretion takes over and is sufficient to maintain pregnancy [25,26]. The contribution of the developing fetus to the generation of progesterone is negligible. Progesterone and its metabolites, dihydroprogesterone (DHP) and allopregnanalone (ALLO), are involved in maintaining healthy pregnancy and protecting the fetus from exposure to harmful levels of maternal glucocorticoids as a result of stress during pregnancy [27]. It has been proposed that one of allopregnanolone’s primary stress-reducing functions is to modulate γ-aminobutyric acid type A (GABA(A)) receptors [28]. Most of what is known about the steroid hormone metabolites during pregnancy or progesterone and its relationship with stress has been discovered through research with animal models [29,30,31,32,33].

**Figure 1:**
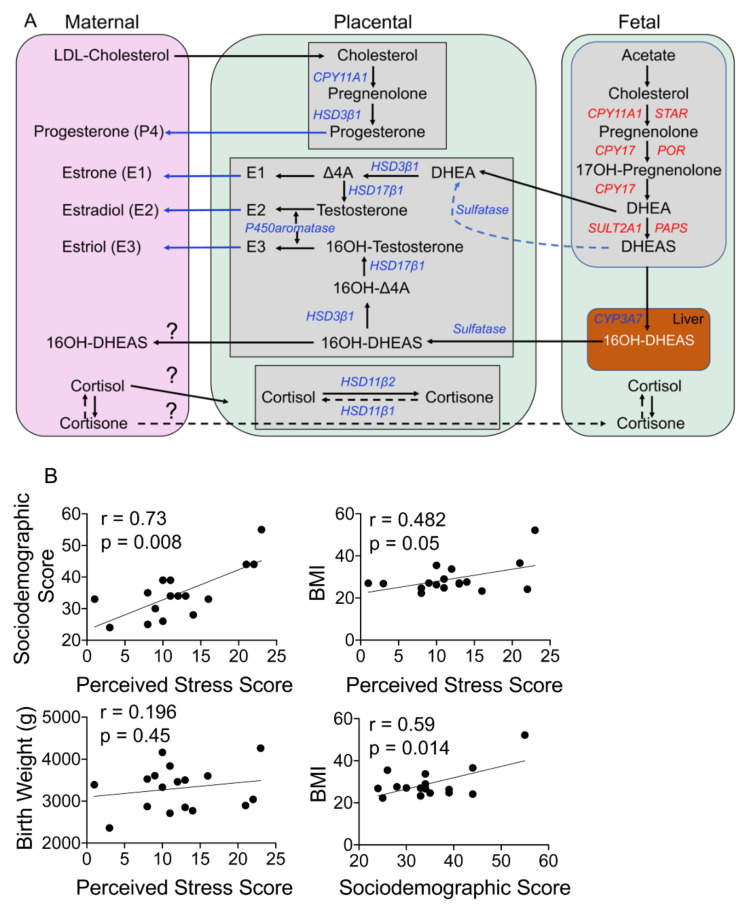
(**A**) Schematic of steroidogenesis in maternal–fetal compartments during pregnancy. Maternal cholesterol is transported to the placenta, where it serves as a precursor for the production of progesterone and its metabolites. Maternal glucocorticoids can be transported to the placenta and across to the fetal circulation. The fetus makes its own cholesterol from acetate and converts it to DHEAS via several steps, as shown. DHEAS is transported to the fetal liver and from there to the placenta, where DHEAS is converted to testosterone and ultimately to estrogens (estrone (E1), estradiol (E2), and estriol (E3)), which are subsequently transported to the maternal circulation from the placenta. Abbreviations: LDL-cholesterol: low-density lipid cholesterol; CYP11A1: cytochrome P450 family 11 subfamily A member 1; CYP17: cytochrome P450 17; CYP3A7: cytochrome P450 family 3 subfamily A member 7; Δ4A: delta 4 androstenedione; DHEA: dehydroepiandrosterone, DHEAS: dehydroepiandrosterone sulfate; HSD3β1: hydroxysteroid dehydrogenase 3-beta isomerase 1; HSD17β1: hydroxysteroid 17-beta dehydrogenase 1; HSD11β1: hydroxysteroid 11-beta dehydrogenase 1; HSD11β2: hydroxysteroid 11-beta dehydrogenase 2; PAPS: 3′-phospho-5′-adenylyl sulfate; POR: P450 oxidoreductase; STAR: steroidogenic acute regulatory protein; SULT2A1: sulfotransferase family 2A member 1. (**B**) Maternal stress associates with her health outcomes but does not affect the baby’s birth weight. Linear regression analysis revealed that the maternal perceived stress score (PSS) correlates positively with her sociodemographic score (*r* = 0.73, *p* = 0.0008) and body mass index (BMI) (*r* = 0.48, *p* = 0.05) but not the infant’s birth weight. The mother’s sociodemographic score positively correlates with her BMI (*r* = 0.59, *p* = 0.014). *N* = 17.

The placenta also becomes the primary source of estrogens approximately by 9 weeks of gestation. As pregnancy progresses, estrone (E1), estradiol (E2), and estriol (E3) concentrations increase in the plasma. Abnormally high E2 levels are associated with first-trimester pregnancy loss. The placenta also synthesizes estriol from dehydroepiandrosterone sulphate (DHEAS) using androgens as intermediary substrates and involving several steroidogenic enzymes [34]. DHEAS made in the maternal and fetal adrenal glands is first transported to the liver, converted to 16a-hydroxy-DHEAS, which is, in turn, transported to the placenta, where it is then made into estriol (Figure 1A). A sudden surge in estriol levels can lead to preterm birth, and estriol is the dominant estrogen at the time of birth, with its levels coinciding with labor onset.

Labor and delivery are the culmination of these important events, with the immediate postpartum period being a critical period for both the mother and the newborn baby. No human study has systematically examined the levels of glucocorticoids, progesterone, estrogen, and their metabolites in maternal and fetal samples side by side postpartum, all of which can be affected by maternal stressors. Nor has any study evaluated the relationship of these hormone levels to maternal stress or birth outcomes. In this study, we determined the levels of 10 different steroid metabolites from maternal (plasma and hair) and fetal (cord blood and placenta) samples. We also determined the associations of these levels with maternal perceived stress status and sociodemographic status, as well as infant birth weight and gestational age.

## 2. Materials and Methods

### 2.1. Study Participants

Nulliparous women (non-smokers only; *n* = 17) were recruited from obstetric clinics at the University of California San Francisco (UCSF) Mission Bay Hospital under the UCSF’s institutional review board protocol #16-10957. The study (i) recruited women during their third trimester of pregnancy when questionnaires regarding sociodemographic and perceived stress were completed and hair samples acquired, (ii) collected their placenta, cord blood, and peripheral maternal blood at delivery, and (iii) acquired data from their medical records on infant birth weight and gestational age.

At the clinic visit, the research assistant provided eligible women with a handout about the project. If interested, the women provided full informed consent. For inclusion, the women had to be 18 years or older, had to speak either English or Spanish, and had to consent (at the minimum) to filling out questionnaires and the collection of placental tissue. Mothers with a history of adrenal illness or endocrine problems, who were on steroids such as prednisone, who smoked, or who had cognitive impairment, were excluded. Fifty-two first-time expectant mothers consented to participate in this study. However, only 17 women completed the perceived stress score (PSS) questionnaire and 3 women wanted their placenta returned for encapsulation, and hence we did not collect the placenta from these 3 women, whereas the placenta from 2 women couldn’t be collected at the time of delivery due to logistic issues. From this cohort, samples were collected, as consented, including placental tissue (*n* = 12), maternal hair (*n* = 7), cord plasma (*n* = 6), and maternal plasma (*n* = 6); not all women consented to donate all 4 samples. Self-report data on sociodemographic information and perceived stress score (PSS; [35]) were collected using questionnaires from all 17 women. In addition, data on gestational age and birth weight (*n* = 17) were determined from each patient’s available medical records.

### 2.2. Questionnaire-Based Measures

The perceived stress scale (PSS) is the most widely used psychological instrument for measuring the perception of stress [35]. It is a measure of the degree to which situations in one’s life are appraised as stressful over the past month. The PSS-10 was designed for use in community samples with at least a junior high school education. The questions are of a general nature and relatively free of content specific to any subpopulation group. The measure has excellent validity and reliability across cultures. The total score was analyzed.

The sociodemographic questionnaire was a self-report questionnaire that acquired information about the women’s age, education, employment status, relationship status, income, insurance coverage, and other important sociodemographic variables. It was used to obtain descriptive data.

## 3. Biological Measures

### 3.1. Collection of Biospecimens 

A small hair sample was collected from the back of each woman’s head, very close to the scalp, and stored for future use at room temperature in an individual zip bag, protected from light. Hair has a predictable growth rate of ~1 cm/month, with the most proximal 1 cm segment to the scalp equating to the past month’s hormonal production and the next 1 cm to the month before that and so on. Up to 3 cm (~3 months) can be used reliably. Anything distal to that has been found to be less reliable due to hormone loss from hair. None of the women had had any hair treatment. Their placentae were stored at 4 °C in the delivery room and collected by one of us (B.H.) within 2–12 h, depending on the time of delivery; maternal peripheral and fetal cord blood samples were acquired at the time of delivery and processed within 2–12 h of delivery. Blood samples were collected in EDTA purple-topped tubes and processed for plasma for metabolomics analyses. All samples were cryo-preserved or stored at −80 °C, as appropriate, for use in future experiments and studies.

### 3.2. Analysis of Steroid Metabolites in Placenta and Cord Blood Using Ultra-Pure Liquid Chromatography (UPLC) Mass Spectrometry (MS)

Steroids from plasma, tissue (15 mg), and hair samples were extracted in anti-oxidant solution (0.2 mg/mL of butylated hydroxytoluene (BHT)/EDTA solution in 1:1 methanol:water) by homogenizing in GenoGrinder 2 × 30 s, centrifuged, followed by washes in appropriate solvents [36], and lyophilized. Samples were reconstituted for liquid chromatography–mass spectrometry (LC-MS) in 100 µL of 1 µM 1-phenyl 3-hexadecanoic acid urea/1-cyclohexyluriedo-3-dodecanoic acid (PHAU/CUDA) in methanol/acetonitrile (50:50) solvent, mixed, and then sonicated for 5 min, followed by centrifugation for 5 min in spin filters, and the supernatant was transferred to glass inserts in a high-performance liquid chromatography HPLC vial. Tritiated steroids were added to monitor the retention time and steroids recovered through HPLC, while deuterated internal standards were added to allow quantification of the compound of interest. To calculate the quantity of the steroid metabolite in each fraction, the area under the peak of the sample was divided by the area under the peak of the deuterated internal standard and represented as pg/mg tissue or in ng/mL for plasma levels.

### 3.3. Statistical Analysis

Linear regression or Spearman correlations (when *n* < 12) were used to compare hormone levels to all other variables. The Mann–Whitney U test was used to compare hormone levels among blood samples and among tissue samples separately. Non-parametric tests were used due to a small sample size. Analyses were computed using Prism software (GraphPad Software Inc., San Diego, CA, USA). A *p*-value <0.05 was considered statistically significant.

## 4. Results

### 4.1. Study Population Characteristics and Steroid Profiles in Maternal and Fetal Samples

Characteristics of the study population are presented in Table 1. In our cohort, one woman had an early-term delivery at 37 weeks, four had late-term delivery (41 weeks, 0 days to 41 weeks, 6 days), whereas 12 women delivered at full term (39 weeks, 0 days to 40 weeks, 6 days). The mothers’ body mass index (BMI), mode of delivery, infant weight, and sex are reported; four women had a BMI of >30, but that did not affect gestation age. During pregnancy, the placenta is the main source of several steroid hormones found in the maternal circulation, including progesterone and estrogens (Figure 1A). Mass spectrometry is a sensitive and reliable method to detect and differentiate between different steroid metabolites simultaneously in a sample; it is well established that the steroid concentrations reported using mass spectrometry are lower than those reported with traditional ELISA or immune assays. We were able to ascertain levels of glucocorticoids, progesterone, and estrogen steroid metabolites and their precursors in maternal and fetal samples; however, several of these metabolites levels were below levels of detection (LOD) in maternal and fetal samples (Table 2). Progesterone was the only metabolite detected in all four different samples analyzed (maternal plasma and hair and fetal cord blood and placenta; Table 2). One woman had cord and plasma steroid metabolite levels that were several-fold higher than the group average; her values are shown separately in Table 2. Maternal perceived stress scores displayed a strong correlation with their sociodemographic score (*r* = 0.73, *p* = 0.0008) and BMI (*r* = 0.48, *p* = 0.05) but not the fetal birth weight; the sociodemographic score also showed a positive correlation with the BMI (*r* = 0.59, *p* = 0.014; Figure 1B).

### 4.2. Maternal or Fetal Cortisol and Cortisone Do Not Correlate with the Maternal PSS

**Figure 2:**
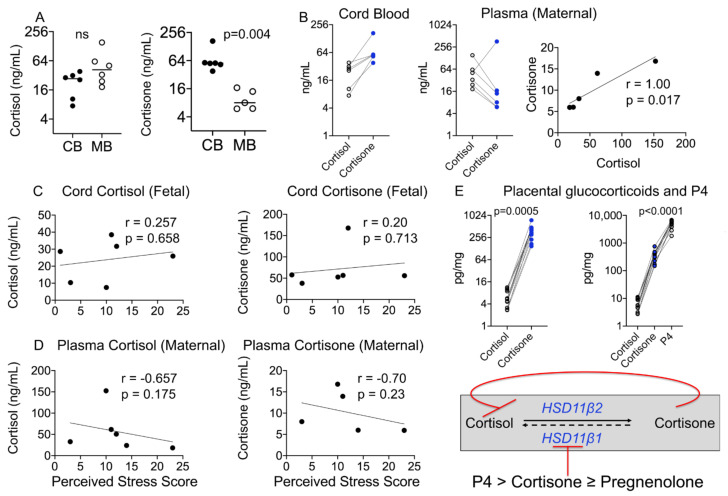
Maternal–fetal glucocorticoid metabolite levels and correlation with birth outcomes and the PSS. (**A**) Maternal–fetal cortisol levels did not differ significantly, but cord cortisone levels were significantly higher than maternal levels (*p* = 0.004). (**B**) Individual cord cortisone levels were higher than cortisol levels, whereas maternal cortisone levels were lower than cortisol levels in all individuals except one. Maternal plasma cortisol correlated positively with maternal plasma cortisone (*r* = 1.00, *p* = 0.017). (**C**) Fetal cortisol or cortisone and (**D**) maternal cortisol or cortisone did not correlate with the PSS. *N* = 6. (**E**) Fetal cortisone levels are maintained high as placental cortisone and progesterone inhibit the activity of HSD11β1; individual placental cortisone levels were higher than placental cortisol levels (*p* = 0.005) and progesterone (P4) levels were ~13-fold higher than cortisone levels (*p* < 0.0001). *N* = 17. Mann–Whitney U test and Spearman’s correlation analysis.

Maternal cortisol plays a crucial role in pregnancy, and stress-induced changes in its levels could interfere with labor and birth outcomes. Cortisol is converted to its inactive metabolite, cortisone, in both maternal and fetal compartments by the actions of the enzyme hydroxysteroid 11β dehydrogenase 2 (HSD11β2), and inactive cortisone can be converted back to cortisol by the action of HSD11β1 (Figure 1A). In our study, cortisol levels tended to be higher in maternal blood compared to cord blood, whereas cord blood cortisone levels were significantly higher than those in maternal blood (Figure 2A). In cord blood, cortisone levels were higher than cortisol levels in maternal samples, whereas this relationship was reversed in maternal blood, with the exception of one individual in the maternal pool (Figure 2B). In maternal blood, there was a strong significant relationship between cortisol and cortisone levels (*r* = 1.00, *p* = 0.017), which was not present in the cord blood samples (Figure 2B).

Cortisol and cortisone are believed to be markers of maternal stress [37,38]; however, we did not find a significant relationship between maternal or fetal cortisol or cortisone levels and the PSS (Figure 2C,D). A strong negative relationship was seen between maternal plasma cortisone levels and the PSS (*r* = −0.70), although this did not reach statistical significance probably due to a small sample size. Because of the diurnal rhythm and rapid change in plasma or salivary concentrations of cortisol and its metabolites, their reliability as markers of chronic stress has been equivocal. It has been proposed that hair samples reflect cumulative hormone levels integrated across the past 3 months, and thus may more accurately reflect sustained stress levels of glucocorticoids. Contrary to our expectation, neither cortisol nor cortisone levels in maternal hair were above the LOD in our cohort (Table 2).

Maternal cortisol plays an important role in labor and fetal lung maturation. Conversion of placental cortisol to cortisone is important in regulating its level. Placental cortisone and progesterone are known to inhibit the activity of HSD11β1 in the presence of cortisol and nicotinamide adenine dinucleotide (NAD+) [39]. While placental cortisol levels were below the LOD, like in cord blood, placental cortisone levels were higher than cortisol levels for individual samples (Figure 2E). Placental progesterone (P4) levels were several-fold higher than cortisol or cortisone levels and together with cortisone probably inhibited the activity of placental HSD11β1 (Figure 2E).

### 4.3. Progesterone and Its Metabolite Levels in Fetal and Maternal Samples

**Figure 3:**
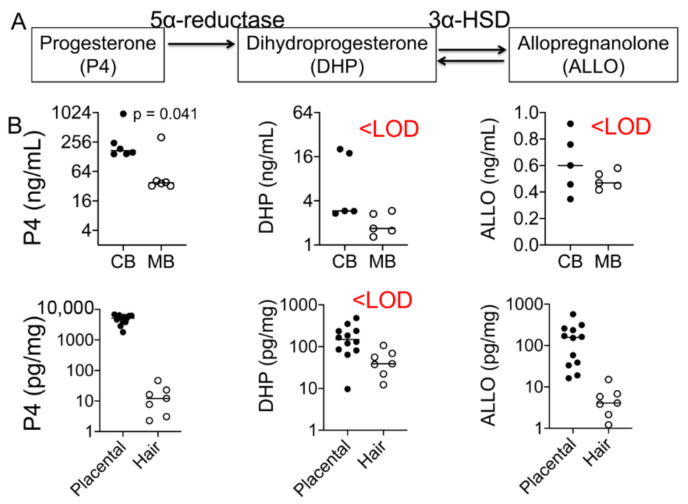
Progesterone and its metabolites’ levels in maternal and fetal samples. (**A**) Graphic illustration of the relationship between progesterone and its metabolites, dihydroprogesterone (DHP) and allopregnanolone (ALLO). Progesterone is converted to DHP by the action of the enzyme 5α-reductase. DHP is converted to the second metabolite ALLO by 3α-HSD. (**B**) Cord progesterone levels were ~3-fold higher than maternal plasma progesterone levels (*p* = 0.041; *n* = 6). Placental progesterone levels were several-fold greater than maternal hair progesterone levels (*p* < 0.0001). DHP levels were below the level of detection (LOD) in both maternal and fetal samples, whereas ALLO was only detected in placental tissues (*n* = 17). Mann–Whitney U test and Spearman’s correlation analysis.

Placental progesterone (P4) is the main source of maternal and fetal plasma progesterone levels during pregnancy (Figure 1A), but how maternal and fetal levels differ is not known. Placental progesterone is converted to dihydroprogesterone (DHP), which, in turn, is converted to allopregnanolone (ALLO) in a non-reversible manner (Figure 3A). In this side-by-side study, we found that cord progesterone levels were several magnitudes higher than maternal plasma levels, whereas DHP and ALLO levels were below the LOD in fetal and maternal plasma samples (Figure 3B). Progesterone and ALLO levels were highest in placental samples, whereas DHP, an intermediary metabolite, was not detected in either maternal or fetal samples (Figure 3B and Table 2).

### 4.4. Maternal or Fetal Progesterone and ALLO Levels Do Not Associate with Birth Outcomes

**Figure 4:**
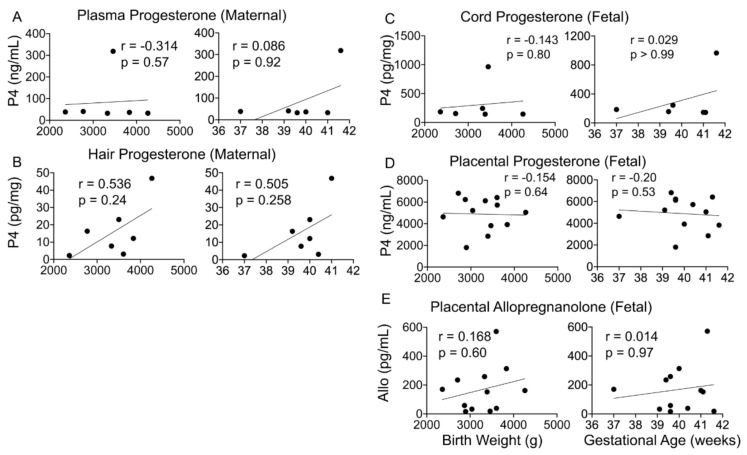
Maternal–fetal progesterone and its metabolites and correlation with birth outcomes. (**A**–**E**) Maternal and fetal progesterone (*n* = 6) or placental ALLO (*n* = 17) levels did not significantly correlate with infant birth weight or gestational age. Spearman’s correlation analysis.

Maternal progesterone levels are known to increase with gestational age and plateau in the last few weeks; however, the status of fetal progesterone and its metabolite levels and their relationship with gestational age and birth weight have not been determined. Although we found no significant relationship between fetal or maternal P4 levels and birth weight or gestational age, fetal and maternal P4 levels exhibited largely opposite relationships with these two outcomes (Figure 4A–D). Placental ALLO levels also did not associate with birth outcomes (Figure 4E).

### 4.5. Hair Progesterone Levels Positively Correlate with the Maternal Perceived Stress Score (PSS)

**Figure 5:**
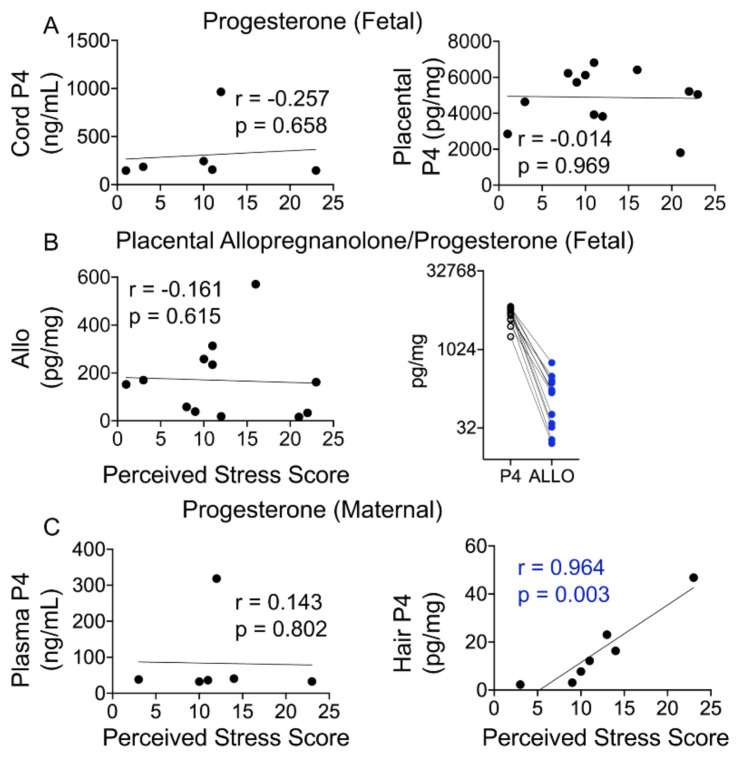
Maternal not fetal progesterone positively correlates with the PSS. (**A**) Cord or placental progesterone levels did not correlate with the maternal PSS. (**B**) Placental ALLO levels also did not correlate with the PSS. Placental progesterone levels were ~29-fold higher than placental ALLO levels, and ALLO levels were lower than P4 in each participant. (**C**) While maternal plasma progesterone levels did not correlate with the PSS, hair P4 levels displayed a significant positive correlation with the PSS (*r* = 0.964, *p* = 0.003). N = 7. Mann–Whitney U test and Spearman’s correlation analysis.

Maternal stress is a major risk factor for preterm labor and is often difficult to quantify. Hair is often seen as a non-invasive way to measure certain metabolite and environmental toxin levels, with the assumption that hair levels reflect what is seen in maternal and fetal circulation. We next ascertained whether maternal and fetal P4 and ALLO levels are associated with the perceived stress score (PSS) and might serve as a more objective measure of stress-related risk. Fetal progesterone or ALLO levels did not exhibit any relationship with the PSS (Figure 5A,B). Although placental ALLO levels did not show any appreciable relationship with the PSS, placental ALLO levels were lower than P4 levels in all individuals (Figure 5B). Hair P4 levels showed a strong positive association with the PSS (P4: *r* = 0.964, *p* = 0.003), but maternal plasma P4 levels did not (Figure 5C).

### 4.6. Maternal Dehydroepiandrosterone Sulfate (DHEAS) Correlates with the Perceived Stress Score

**Figure 6:**
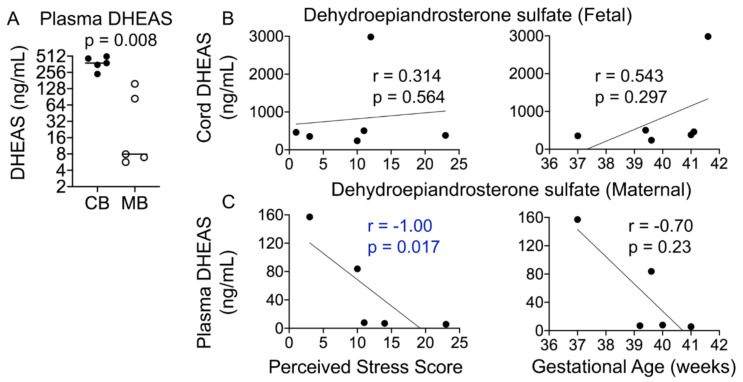
Maternal plasma DHEAS levels negatively correlate with the PSS. (**A**) Cord DHEAS levels were ~7-fold higher than maternal plasma DHEAS levels (*p* = 0.008). (**B**) Cord DHEAS levels did not correlate significantly with the PSS or gestational age. (**C**) Maternal plasma DHEAS levels correlated negatively with the PSS (*p* = 0.017) but not with gestational age. Mann–Whitney U test and Spearman’s correlation analysis.

During pregnancy, DHEAS is made in the developing fetal adrenals and transported to the placenta, where it serves as a precursor for androgens and estrogens; both DHEAS and estrogens in the maternal circulation during pregnancy are thus largely of fetal origin (Figure 1A). Cord DHEAS levels were significantly higher than those in maternal plasma (~7.4-fold, *p* = 0.0008; Figure 6A); however, fetal DHEAS levels did not correlate with gestational age or the PSS (Figure 6B). Interestingly, maternal plasma DHEAS levels correlated negatively with the PSS (*r* = −1.00, *p* = 0.017) and gestational age (*r* = −0.70, *p* = 0.23; Figure 6C).

### 4.7. Fetal Estradiol Correlates with the Perceived Stress Score

**Figure 7:**
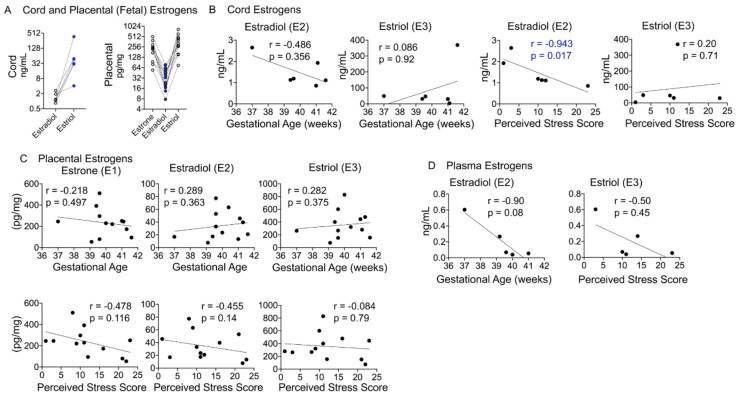
Maternal–fetal estrogen metabolites and correlation with birth outcomes and the PSS. (**A**) Cord estriol levels were ~20-fold higher than estradiol levels and increased in each individual. In the placenta, estrone and estriol levels were higher than estradiol levels, which were lowest in each participant. (**B**) While neither cord E2 nor E3 levels correlated with gestational age, E2 levels were significantly negatively correlated with the PSS (*r* = −0.94, *p* = 0.017). (**C**) Placental estrogens did not correlate with gestational age or the PSS. (**D**) Plasma E2 levels showed a strong negative correlation with gestational age, with a trend toward significance (*r* = −0.90, *p* = 0.08). Mann–Whitney U test and Spearman’s correlation analysis.

Estrogen metabolites are synthesized from DHEAS and androgens in the placental compartment and exported to the maternal circulation (Figure 1A). In our cohort, all three estrogen metabolites (estrone (E1), estradiol (E2), and estriol (E3)) were detected in the placental compartment, E2 and E3 were detected in cord blood, and only E2 was quantifiable in the maternal plasma (Table 2). Estriol levels were higher than estradiol levels in cord blood, whereas estrogen metabolites displayed a V-shaped relationship in the placental samples (Figure 7A). Cord estradiol levels did not correlate with birth outcomes but showed a significant negative relationship with the PSS (*r* = −0.943, *p* = 0.017), whereas estriol levels did not show a significant correlation with either birth outcomes or the PSS (Figure 7B). Placental estrogens also did not show a significant correlation with either birth outcomes or the PSS (Figure 7C). Maternal estradiol levels showed a strong negative correlation with gestational age that trended toward significance (*r* = −0.90, *p* = 0.08), but not with the PSS (Figure 7D).

## 5. Discussion

Experiencing too much stress from negative life events or other stressors during pregnancy can have serious consequences for the pregnant mother and the baby in the womb, including postnatal depression, preterm birth, low birth weight, and child developmental/behavioral problems [1,2,3,7,40,41,42,43,44,45]. In addition to the personal suffering caused by these negative outcomes, they constitute growing public health concerns that are associated with huge economic costs (preterm births alone cost at least $26 billion annually). The Centers for Disease Control administers an annual population-based pregnancy risk surveillance system (PRAMS). The data for 2000–2010 show that three quarters of women experience at least one stressful life event in the year before their infant’s birth [16]. Stressors are most frequently financial, followed by emotional/partner-related stressful events; they are especially prevalent among women of low socioeconomic status and women covered by Medicaid. Most women, however, including those of higher socioeconomic status, report stress during pregnancy. The American College of Obstetricians and Gynecologists recommends that all pregnant women, regardless of socioeconomic status, receive screening for psychosocial complications during their prenatal visits [46]. To more precisely understand the risk that stress creates for healthy pregnancy, a study of potential biological factors is essential. The goal of this study was to examine a set of steroid hormone metabolites that are essential for maintaining a healthy pregnancy and whether maternal perceived stress alters the levels of these steroid hormones in the maternal or fetal or both compartments.

In this study, we simultaneously determined concentrations of 10 different steroid hormone metabolites using mass spectrometry in maternal and fetal samples obtained immediately postpartum. While the mother’s perceived stress score was positively associated with her sociodemographic score and both measures were also positively associated with the maternal BMI, neither maternal measure affected the fetal birth weight or gestation age. The relative concentrations of steroid hormone metabolites (cortisol vs. cortisone, estradiol vs. estriol, progesterone vs. ALLO, etc.) rather than their absolute levels are important for maintaining a healthy pregnancy, as ascertained from studies using maternal–fetal samples in non-human primates [33,39,47] or human studies of preterm birth [6]. Here, we report that in healthy human pregnancies, the relative concentrations of these steroid metabolites are maintained, irrespective of the maternal perceived stress score, suggesting that maternal stress is effectively buffered by a healthy placenta. The placenta responds to maternal cortisol levels and forms part of the body’s stress response system, so the effects of any stress that the fetus experiences are present in the placenta. Stress may result in dysregulation of placental biology, with potential effects on biochemical and metabolic pathways that may disrupt the placental clock and hormonal balance based on the degree of stress [7,8]. Problems with the placenta are known to lead to conditions such as gestational diabetes, preeclampsia, fetal growth restriction, preterm birth, and stillbirth [9,10,11,12,13,14,15]. But the mechanisms responsible for healthy versus problematic outcomes from stress are not completely understood.

In agreement with other studies that have recently reported levels of steroid hormones in pregnant women using similar mass spectrometry methodologies [48] or radio-immunoassays [23,49], concentrations of most hormones reported in this study are lower than previously reported methods that employ either direct or indirect immunoassays. Hair cortisol is often cited as a surrogate for predicting maternal stress levels or a marker of hypothalamic–pituitary–adrenal axis function in pregnant women [50,51,52,53]; however, hair cortisol levels reported in many of these studies were measured using salivary cortisol immunoassays. In our study, hair cortisol and cortisone levels were below the levels of detection; in fact, progesterone was the only steroid hormone that was above the limit of detection in hair samples. None of the women in our cohort used any hair dye or treatments that would confound results. Thus, quantification of steroid hormones in maternal hair remains a challenge.

### 5.1. Relationships among Maternal and Fetal Hormones

There is an exchange of hormones between the mother and the fetus during pregnancy (Figure 1A). Comparing levels in each of the tissues we sampled provides an idea of how that transfer may occur in terms of each metabolite. Since the placenta is the main site for the synthesis of progesterone and estrogens during pregnancy, not surprisingly, the levels of these steroid hormones were highest in the placental compartment (Table 2). Progesterone levels were found to decrease from the placenta to cord blood to maternal blood to hair. Although hair P4 levels were lower than in maternal blood and often exhibited an opposite relationship with our outcome measures compared to blood levels of progesterone, at least toward the end of pregnancy (weeks 37–41). As noted earlier, hair and plasma measure different time periods in their assessment of hormone levels (i.e., chronic versus more acute stress levels). Thus, they may provide uniquely different types of information about hormone levels. Further research is needed to understand these potentially valuable distinctions.

In current practice, maternal hair and/or blood are used to predict fetal outcomes. However, our findings show that trends in the mother do not necessarily resemble those found in the fetus. For example, fetal progesterone levels correlated negatively with birth weight and gestational age. In addition, maternal hair progesterone levels were positively correlated with birth weight and gestational age, although neither relationship reached statistical significance. Our results suggest that care must be taken when assuming that one tissue marker will accurately reflect another. Maternal and fetal tissues as well as differing tissues within a mother or fetus appear to provide unique indicators of risk, which must be established for each biomarker or metabolite separately.

Decreased serum progesterone levels or antagonism of progesterone receptor function is known to cause preterm labor and birth. Progesterone may result in remodeling of the cervix, critical for parturition [54]. Initial analysis of data from the OPPTIMUM study, a randomized controlled clinical trial that sought to evaluate the efficacy of vaginal progesterone for prevention of preterm birth and associated adverse perinatal outcomes in singleton pregnancies with a short cervix, did not find a significant effect of progesterone treatments on prevention of preterm birth [55]. The study reported no long-term benefit or harm of progesterone treatment. Subsequent meta-analysis of aggregate data found that vaginal progesterone significantly reduced the risk of preterm birth at ≤34 weeks or fetal death by 34% in a subset of pregnant women [56].

### 5.2. Glucocorticoid Metabolites as Markers of Stress in a Healthy Pregnancy

Cortisol and cortisone are believed to be reliable markers of stress; yet, when levels in maternal and fetal tissues are compared to perceived stress, they showed no correlation. While cortisol may be a marker of temporary changes in stress, the levels possibly fluctuate too much to hold a significant correlation with more long-term perceived stress or sociodemographic status. Cortisol and cortisone did not show a correlation with the BMI, gestational age, or birth weight either in our cohort. Unlike previous reports, we were unable to reliably measure cortisol or cortisone levels in maternal hair obtained in the third trimester. The difference in cortisol levels can only be explained due to the use of less sensitive immunosorbent assays. This does not rule out that hair may still be reliably used to predict the concentrations of other environmental toxins or tobacco metabolites such as cotinine.

Maternal cortisol plays an important role in labor as well as fetal lung maturation. Mice with glucocorticoid receptor knockout are embryonic lethal, and mice with corticotropin-releasing factor knockout, an upstream regulator of glucocorticoid synthesis and release from the adrenals, fail to deliver pups that survive. Thus maternal–fetal glucocorticoid synthesis and levels are tightly regulated. Placental glucocorticoids can perturb this relationship, and studies on both human and baboon placenta have shown that placental progesterone is a potent inhibitor of HSD11β1 activity and no conversion of cortisone to cortisol is measurable. Besides progesterone, cortisone and pregnenolone also inhibit HSD11β1 activity up to 50%, whereas estrogens and other steroid metabolites do not [39]. In agreement with these studies, we found undetectable levels of cortisol in the placenta, thereby suggesting that in a normal, healthy pregnancy, placental HSD11β1 activity is suppressed by the presence of high levels of P4 and cortisone.

### 5.3. Progesterone and Its Metabolites Correlate with Perceived Stress in a Healthy Pregnancy

Maternal progesterone levels at term are reported in the range of 150–175 ng/mL, whereas fetal P4 levels are reported to be 2–3-fold greater [6]. The average progesterone levels in our cohort in maternal plasma immediately postpartum were lower than those reported; however, fetal P4 levels were nearly 4.8-fold greater (36.28 ± 1.64 vs. 175.66 ± 18.67 ng/mL) in agreement with previous findings. In our study, progesterone and its metabolites in fetal tissue (placenta and cord blood) did not correlate with maternal perceived stress, suggesting that in a healthy pregnancy with a healthy placenta, the mother’s perceived stress does not appreciably alter fetal steroidogenesis. We found no relationship of P4 or ALLO levels with stress in these tissues. However, hormone levels in maternal tissue were associated with maternal stress. In particular, P4 levels in hair were positively associated with greater perceived stress in the last trimester. Thus, a woman’s perceived stress may not be transmitted to the fetus in significant ways, at least in a healthy and term pregnancy. This is important in showing that although maternal stress may affect maternal levels of these hormones, the fetus may remain largely unaffected by maternal stress, at least from glucocorticoid, progesterone, and estrogen metabolites. Clearly, these findings need to be replicated in a bigger cohort.

### 5.4. Progesterone and Its Metabolites as Predictors of Birth Outcomes

In otherwise healthy and uncomplicated pregnancies, P4 levels did not appear to be a useful predictor of either gestational age or birth weight, as evidenced in any tissue sample. Progesterone supplements have been used as a prevention measure for preterm birth or miscarriages; however, our data raise questions regarding whether higher maternal progesterone levels actually result in better birth outcomes for the fetus. DHP and ALLO levels, metabolites of progesterone, were below the levels of detection in maternal samples, whereas placental ALLO levels were lower than placental P4 levels for all individuals and nearly 29-fold less than P4 levels. In light of these findings, a systematic determination of progesterone and ALLO may help understand whether these analytes are associated with gestational age and possibly preterm birth.

### 5.5. Estrogen Metabolites and Their Relationship with Birth Outcomes and Maternal Stress

The placenta serves as the primary source of estrogens and progesterone metabolites as gestation ensues. Other studies have reported concentrations of estrone, estradiol, and estriol in maternal plasma in different trimesters [48]. Mean estradiol concentrations at abnormally high estradiol levels are associated with first-trimester pregnancy loss. In baboons, administration of a highly specific nonsteroidal aromatase inhibitor, CGS 20,267 that causes maternal estrogen levels to fall to <0.1ng/mL but does not alter progesterone levels, resulted in 50% loss of pregnancy, which was completely prevented by exogenous estradiol administration alone [47]. Estriol is the dominant estrogen at the time of birth, with its levels coinciding with labor onset. While we were unable to detect estriol in maternal samples immediately postpartum, placental estriol levels were higher than E1 or E2 levels at the time of birth. Furthermore, cord estradiol levels correlated negatively with the mother’s PSS, and maternal estradiol levels were negatively associated with gestation age, as has been shown before [7,8,37]. These data suggest an interplay between maternal stress, androgens, and placental steroidogenesis. Our data also suggest that maternal estradiol and estriol may be worth investigating in larger studies that include women with preterm birth or complications of pregnancies.

## 6. Conclusions

In conclusion, relative levels of steroid hormone metabolites rather than absolute levels maintain a healthy pregnancy. A woman’s stress alters her circulating steroid hormone metabolites, and the placenta serves as an effective barrier and possibly buffers the developing fetus from fluctuating hormonal changes that might occur in the mother with exposure to various stressors.

## Figures and Tables

**Table 1:** Study population characteristics.

Patient	Gestational Age	BMI	Infant Weight (g)	Infant Sex	Apgar Score	Mode of Delivery
P-002	39 + 4	24.8	2710	F	9 9	NSVD
P-006	39 + 2	27.6	2772	F	9 9	NSVD
P-016	39 + 6	26.3	3330	M	9 9	NSVD
P-022	37 + 0	26.9	2360	F	9 9	NSVD
P-023	41 + 3	23.3	3600	M	8 9	NSVD
P-026	41 + 1	27.0	3390	M	8 9	NSVD
P-030	40 + 0	26.7	3500	F	8 9	NSVD
P-032	40 + 0	29.0	3835	F	9 9	LTCS
P-034	41 + 0	52.2	4260	F	7 9	NSVD
P-037	39 + 3	24.7	3525	M	9 9	VAVD
P-038	41 + 6	33.8	3460	F	8 9	NSVD
P-041	39 + 6	36.6	2895	F	4 9	NSVD
P-043	40 + 4	27.1	3605	F	8 9	NSVD
P-046	39 + 1	27.1	2850	F	1 9	LTCS
P-048	39 + 6	22.3	2870	F	4 9	NSVD
P-049	39 + 1	24.2	3040	M	9 9	NSVD
P-051	39 + 3	35.5	4160	M	3 8	LTCS

Apgar score: appearance, pulse, grimace, activity, and respiration score; BMI: body mass index; LTCS: low transverse cesarian section; NSVD: normal spontaneous vaginal delivery; VAVD: vacuum-assisted vaginal delivery.

**Table 2:** Steroid metabolite concentrations in maternal and fetal samples.

Metabolite	LOD (ng/mL)	Cord Plasma ± SEM (ng/mL)		Maternal Plasma ± SEM (ng/mL)		LOD (pg/mg)	Placenta Mean ± SEM (pg/mg)	Hair Mean ± SEM (pg/mg)
		Patient X	Average ± SEM	Patient X	Average ± SEM			
Cortisol (CRTL)	1.811	31.68	22.18 ± 5.82	50.68	57.96 ± 24.81	36.22	<LOD (<7.06)	<LOD (<0.76)
Cortisone (CRTN)	0.360	167.78	52.21 ± 3.62	361.72	10.15 ± 2.21	7.20	370.82 ± 47.05	<LOD (<0.98)
Progesterone (P4)	0.393	965.64	175.66 ± 18.67	318.46	36.28 ± 1.64	7.86	4884.46 ± 443.97	15.92 ± 5.85
Allo-pregnanolone (ALLO)	4.774	1.02	<LOD (<0.72)	12.3	<LOD (<0.50)	95.48	168.88 ± 46.73	<LOD (<7.31)
Dihydroprogesterone (DHP)	39.530	24.44	<LOD (<13.33)	5.59	<LOD (<2.32)	790.60	<LOD (<200.00)	<LOD (<60.88)
Estrone (E1)	2.702	4.99	<LOD (<3.3)	69.22	<LOD (<0.32)	54.03	232.78 ± 37.47	<LOD (<1.28)
Estradiol (E2)	0.680	1.11	1.55 ± 0.33	18.27	3.96 ± 0.56	13.61	34.32 ± 6.29	<LOD (<0.12)
Estriol (E3)	2.882	369.04	32.28 ± 8.04	674.53	<LOD (<1.72)	57.64	356.34 ± 61.12	<LOD (<0.83)
Dehydroepiandrosterone (DHEA)	7.205	0.407	<LOD (<0.30)	0.03	<LOD (<0.48)	144.10	<LOD (<1.08)	<LOD (<2.25)
Dehydroepiandrosterone sulfate (DHEAS)	18.424	2983.48	387.47 ± 45.83	4969.63	52.28 ± 30.13	368.49	<LOD (<1.53)	<LOD (<4.39)

Steroid metabolite values in fetal (cord plasma and placenta) and maternal (plasma and hair) tissue samples. Plasma values are shown in ng/mL and tissue values in pg/mg. Limit of detection (LOD) values for plasma and tissue are shown. One patient (X) had cord and maternal plasma values several-fold higher than the group average and are shown in a separate column. Her placental and hair values did not differ from the group average and are hence not shown. This patient’s values were not excluded from the analyses, as excluding her cord or plasma values did not significantly change the results, except for the maternal cortisol:cortisone relationship. SEM: Standard Error of Mean.

## Data Availability

Data is contained within the article.
